# Comparative Evaluation of the Mechanical Properties of an Alkasite-Based Material and a Conventional Resin Composite

**DOI:** 10.3390/polym18212788

**Published:** 2026-11-08

**Authors:** Selma Jakupović, Damir Duratbegović, Nedim Pervan, Enis Muratović, Vedran Jakupović

**Affiliations:** 1 Department of Restorative Dentistry and Endodontics, Faculty of Dentistrywith Dental Clinical Center, University of Sarajevo, 71000 Sarajevo, Bosnia and Herzegovina; jakupovic_selma@yahoo.com; 2 Department of Pediatric and Preventive Dentistry, Faculty of Dentistry with Dental Clinical Center, University of Sarajevo, 71000 Sarajevo, Bosnia and Herzegovina; damirduratbegovic@gmail.com; 3 Department of Mechanical Design, Faculty of Mechanical Engineering, University of Sarajevo, 71000 Sarajevo, Bosnia and Herzegovina; muratovic@mef.unsa.ba; 4 Department of Community Healthcare, Faculty of Health Studies, University of Sarajevo, 71000 Sarajevo, Bosnia and Herzegovina; vedran.jakupovic@fzs.unsa.ba

**Keywords:** alkasite, Cention Forte, resin composite, Vickers microhardness, bottom-to-top ratio, diametral tensile strength, compressive strength, filler content, curing mode

## Abstract

Alkasites are resin-based restorative materials that differ from conventional resin composites in the composition and reactivity of their filler system, which enables ion release. Whether optional light activation changes the mechanical properties of the alkasite Cention Forte, compared with self-curing and with a conventional composite, remains insufficiently documented. This in vitro study compared Cention Forte (urethane dimethacrylate-based matrix; reactive calcium fluorosilicate and barium glass fillers) in self-cured (G1) and light-cured (G2) modes with the nanohybrid composite Tetric EvoCeram (G0; dimethacrylate matrix; barium glass, ytterbium trifluoride, mixed oxide and prepolymerized fillers). Top- and bottom-surface Vickers microhardness (VMHt, VMHb), bottom-to-top ratio (BTR), diametral tensile strength (DTS) and compressive strength (CS) were measured (*n* = 7). G1 showed the highest VMHt (70.3 HV), followed by G0 (63.0 HV) and G2 (57.8 HV) (*p*< 0.001). Both Cention Forte groups had BTR values of approximately 1.00 (G0: 0.97) as an indirect, surface-based index. G2 exhibited the highest DTS (39.7 MPa) and G0 the highest CS (311.2 MPa). Curing mode accounted for most between-group variance in microhardness, whereas material composition dominated compressive strength.

## 1. Introduction

Resin-based composites are among the most widely used direct restorative materials because of their favorable esthetic properties, adequate mechanical performance, and versatility in clinical applications. Their composition consists of three main components: an organic matrix containing bifunctional monomers such as bisphenol A-glycidyl methacrylate (Bis-GMA), urethane dimethacrylate (UDMA), and triethylene glycol dimethacrylate (TEGDMA) [1,2]; inorganic fillers, including silicate glass, quartz, aluminum silicate, ceramic particles, yttrium and ytterbium trifluorides, and prepolymerized particles [3,4,5]; and an interfacial coupling agent, usually silane. Other components include color stabilizers, pigments, photoinitiators, inhibitors, and radiopaque agents. Upon exposure to curing light, photoinitiators initiate the polymerization reaction, converting organic monomers into a polymer network [3].

Conventional sculptable resin composites are generally polymerized in increments of up to 2 mm to ensure adequate light penetration and polymerization throughout the material. According to the reciprocity law of exposure, delivering an energy density of 16 J/cm^2^ may be achieved by applying light at an intensity of 400 mW/cm^2^ for 40 s, 800 mW/cm^2^ for 20 s, or 1200 mW/cm^2^ for approximately 13 s [3]. To shorten clinical procedures, contemporary dental materials have introduced bulk-fill composites, which can be placed in increments of 4 mm or more, alongside high-intensity light-curing units (>2000 mW/cm^2^). Such curing units are reported to polymerize 4-mm-thick bulk-fill increments within 3–5 s, depending on the material and curing protocol [6,7].

Nevertheless, conventional resin composites have inherent limitations, including polymerization shrinkage, polymerization-induced stress, and limited ion release and acid-neutralizing capacity. These limitations have stimulated the development of restorative materials with modified formulations designed to combine the mechanical advantages of resin composites with additional functional properties.

Bioactive composites belong to a broader category of bioactive restorative materials that has recently been classified as a distinct group of restorative materials [8]. Bioactive materials release ions, such as calcium, fluoride, phosphate, and hydroxyl ions, as well as monomers and/or particles containing elements such as silver and strontium, to induce specific biological, reparative, or regenerative responses in living tissues, organisms, or cells [9]. A bioactive restorative material should promote a beneficial biological process involved in the repair or replacement of lost tooth structure, stimulate or direct a specific cellular or tissue response, or interact with microorganisms by modulating or suppressing bacterial metabolism [10,11].

Within this broader group of ion-releasing restorative materials, alkasites have emerged as a distinct category of resin-based restorative materials. They contain reactive glass fillers in addition to conventional reinforcing fillers. The reactive glass phase enables the release of ions, including calcium and fluoride, and contributes to acid-neutralizing properties. Thus, alkasites differ from conventional resin composites not because they lack a resin matrix, but because of the specific composition and reactivity of their inorganic filler system. Cention Forte (Ivoclar, Schaan, Liechtenstein) is a commercially available representative of this material category. The manufacturer recommends using a primer to condition the cavity before material placement and enhance adhesion. Cention Forte is self-curing, although its setting reaction can optionally be accelerated by light exposure [12].

The distinctive characteristics of Cention Forte are primarily associated with its inorganic filler system, which contains calcium fluorosilicate glass, calcium-barium-aluminum fluorosilicate glass, barium glass, ytterbium trifluoride, and prepolymerized filler components (isofillers). Its reported inorganic filler content is approximately 78.4 wt% and 57.6–59 vol%. These reactive fillers distinguish the material from conventional resin composites and are considered responsible for its ion-releasing and acid-neutralizing behavior [12,13,14].

The setting reaction of Cention Forte begins with the mixing of its powder and liquid components, initiating a chemically activated polymerization process involving the formation of a polymer network and interaction with silanized glass filler particles. Additional light curing is optional and accelerates polymerization. The combination of chemical and light-activated curing enables bulk placement of the material [13]. According to the reported protocols, curing can be accelerated using a light intensity of 1000 mW/cm^2^ for 15 s or 2000 mW/cm^2^ for 10 s [15]. Studies by Par et al. have demonstrated that Cention exhibits acid-neutralizing capacity [16] and may help prevent enamel [17] and dentin [18] demineralization during prolonged acid exposure. Some authors have further suggested that Cention may promote the remineralization of demineralized dentin and represents a bioactive restorative material with distinctive remineralizing potential [12,19].

In contrast, Tetric EvoCeram (Ivoclar, Schaan, Liechtenstein), also investigated in the present study, is a conventional resin composite. Its resin matrix is based on dimethacrylate monomers, while its inorganic filler system includes barium glass, ytterbium trifluoride, mixed oxide, and prepolymerized filler components. The reported inorganic filler content is approximately 75–76 wt% and 53–55 vol%, depending on the formulation. Tetric EvoCeram is generally applied in increments of up to 2 mm and light-cured at an intensity of 1000–1300 mW/cm^2^ for 10 s, according to the applicable material instructions. Although Cention Forte and Tetric EvoCeram have relatively high filler contents, their filler composition, curing mechanisms, and intended functional characteristics differ [20].

Despite the increasing clinical use of Cention Forte, the evidence on its mechanical behavior is limited and partly contradictory. Most published studies investigated its predecessor, Cention N, and the results for the two products are not always distinguished. In addition, the effect of the optional light activation remains unclear: some studies reported a higher degree of conversion and better mechanical properties after light curing [21,22,23], whereas others found no effect or even lower values than after self-curing [21,22]. Few studies have compared both curing modes of Cention Forte with a conventional nanohybrid composite applied according to its own incremental protocol, using identical specimen geometry and test conditions for several mechanical properties. It therefore remains unknown whether light activation of Cention Forte changes its surface microhardness and strength relative to self-curing, and how both curing modes compare with a conventional resin composite.

Therefore, this in vitro study aimed to compare the Vickers microhardness, bottom-to-top microhardness ratio, diametral tensile strength and compressive strength of Cention Forte in self-curing and light-curing modes with those of the conventional nanohybrid composite Tetric EvoCeram, and to distinguish the effect of curing mode from that of material composition.

## 2. Materials and Methods

The materials used in the study are listed in Table 1. All materials were tested in shade A2, which was available for both materials, to exclude the influence of shade and translucency on light transmission and polymerization.

### 2.1. Research Methods

In this study, previously validated in vitro methods were used to evaluate the mechanical properties of the materials [23,24,25,26,27,28,29,30,31].

The test methods were adopted from previously published in vitro studies [23,24,25,26,27,28,29,30,31]. Vickers microhardness of the top and bottom surfaces of disk-shaped specimens, used to evaluate the curing efficiency of conventional and bulk-fill resin composites under different light-curing intensities, exposure times and light-guide distances, was determined as described by Rizzante et al. [28], Par et al. [30] and Duratbegović et al. [31]; the bottom-to-top microhardness ratio was calculated from these values [28,30]. Diametral tensile strength was determined on disk-shaped specimens loaded along their diameter until fracture, and compressive strength on cylindrical specimens loaded axially until fracture, in a universal testing machine at a constant crosshead speed, following the procedures of Menezes-Silva et al. [23], Kunte et al. [25] and Chandru et al. [26]. Comparable microhardness and compressive strength protocols have also been applied to Cention N by Verma et al. [29], and the specimen preparation and light-curing procedures followed those used for conventional and bulk-fill composites by Par et al. [24] and Attik et al. [27].

The mechanical properties investigated were as follows: Vickers microhardness of the top and bottom surfaces (VMHt and VMHb), the bottom-to-top ratio (BTR), diametral tensile strength (DTS), compressive strength (CS).In addition, the effects of curing mode and material on these properties were separated by planned comparisons (Section 2.2).

All light-cured specimens were polymerized with a Bluephase PowerCure unit (Ivoclar Vivadent, Schaan, Liechtenstein; 385–515 nm; light guide with a 9-mm tip diameter). Tetric EvoCeram (G0) was cured in the High Power program (approximately 1200 mW/cm^2^, 10 s) for each 2-mm increment. Cention Forte G2 was cured in the Turbo program (approximately 2000 mW/cm^2^), which delivers a single 5-s exposure; it was therefore activated twice in succession (2 × 5 s, total exposure 10 s), the second exposure being started immediately after the first without repositioning the light guide. The light guide was held perpendicular to the specimen surface, in contact with the polyester strip covering the top surface (light-tip distance ≈ 0 mm). The irradiance was checked with the radiometer integrated into the curing unit after every seven exposures, and deviations within ±10% of the nominal value were accepted. The same curing protocol was used for all specimen geometries: microhardness and DTS specimens (4 mm; Cention Forte as a single increment, Tetric EvoCeram in two 2-mm increments) and CS specimens (6 mm; Cention Forte as a single increment, Tetric EvoCeram in three 2-mm increments), with curing from the top surface only.

#### 2.1.1. Vickers Microhardness Testing

Vickers microhardness represents the surface hardness of a material and is determined based on the surface area of the indentation produced by a pyramidal indenter in the tested material under a specified load.

Vickers microhardness was measured on the top and bottom surfaces of disk-shaped specimens (4 mm high, 8 mm in diameter) fabricated in a cylindrical silicone mold. The mold was placed on a glass slide covered with a 50-μm polyester strip, filled with restorative material, and covered with a second polyester strip and glass slide. Pressure was applied to condense the material.For the light-cured material groups, polymerization was performed exclusively from the top surface of each specimen.

The alkasite material Cention Forte (Ivoclar Vivadent AG) was placed in the mold as a single 4-mm increment. Group G1 specimens were allowed to self-cure, while G2 specimens were light-cured with two consecutive 5-s exposures in the Turbo program (approximately 2000 mW/cm^2^; 2 × 5 s, total exposure 10 s)the second exposure being started immediately after the first without repositioning the light guide. Tetric EvoCeram (G0) was dispensed from a Cavifil in two 2-mm increments, each light-cured at 1200 mW/cm^2^ for 10 s. All specimens were prepared at room temperature (21–23°C) and a relative humidity of 40–60%.

During preparation, the self-cured specimens (G1) were condensed through the glass slide with a static load of 500 g and left under this load for 6 min to set. The light-cured specimens (G0 and G2) were condensed by manual pressure on the glass slide, the excess material was removed, and the specimens were light-cured immediately. After setting or light curing, the specimens were removed from the molds and stored in distilled, deionized water at 37 ± 1 °C in light-proof containers for 24 h, in the same way as the strength specimens. Microhardness was then measured at room temperature (21–23 °C) and a relative humidity of 40–60%.

Vickers microhardness measurements were performed using an electronic microhardness testing device (QNESS 60 M EVO, ATM Qness GmbH, Mammelzen, Germany). The device was equipped with a Vickers diamond indenter and an optical microscope with magnifications of 5× and 20×.

**Figure 1:**
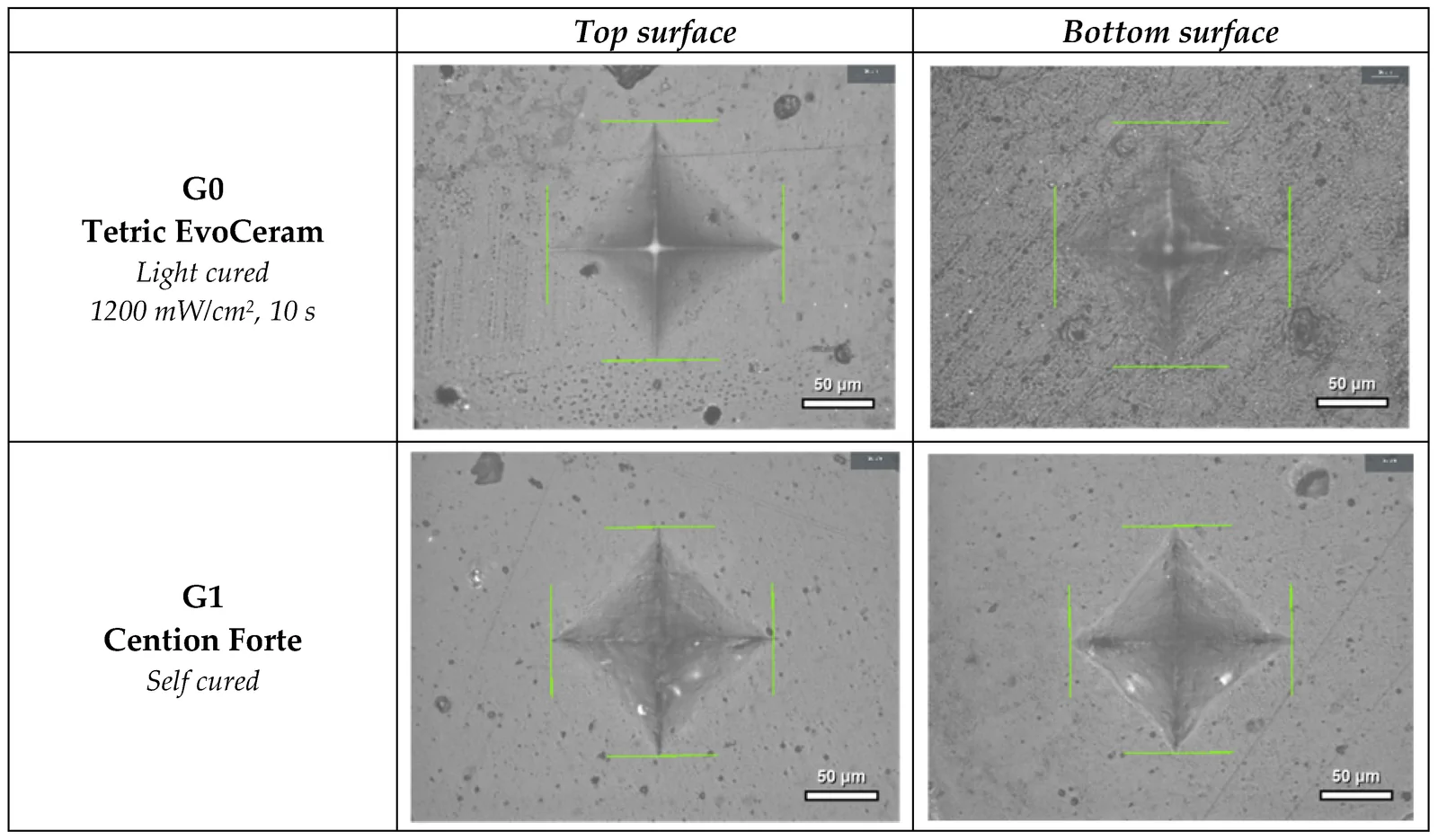

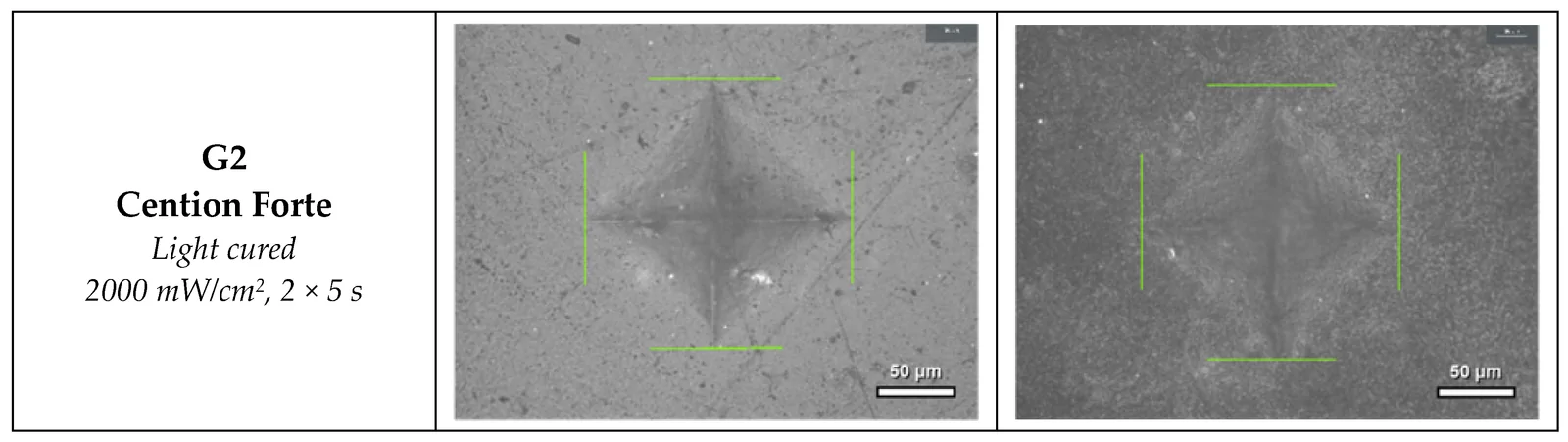
Representative optical micrographs (20× objective of the QNESS 60 M EVO microhardness tester) of Vickers indentations on the top and bottom surfaces of the tested materials. Scale bar: 50 µm.

A test load of 1 kp (1000 g) was applied for 15 s. Vickers microhardness was automatically calculated using the equation:

HV1 = 0.1891 × F/d^2^

where F is the applied load in newtons (N) and d is the mean length of the indentation diagonals in millimeters (mm). Five measurements were taken on each surface, and mean values were calculated for the top (VMHt) and bottom (VMHb) surfaces (Figure 1).The sample size for each experimental group was seven specimens (*n* = 7).

#### 2.1.2. Bottom-to-Top Ratio (BTR)

The degree of polymerization, expressed as the bottom-to-top ratio (BTR), is a parameter used to assess polymerization efficiency. It is derived from the surface microhardness values measured on the bottom and top surfaces of the specimens.

In adequately polymerized resin composite specimens, the difference between the microhardness values of the top and bottom surfaces should not exceed 20%. Bouschlicher et al. showed that a bottom-to-top microhardness ratio of 0.80 corresponds to a bottom-to-top conversion ratio of approximately 0.90 [32]; on this basis, BTR ≥ 0.80 is generally accepted as the criterion of adequate curing, and the effectiveness of light polymerization at the bottom surface of the specimens can be evaluated by determining the ratio of bottom-to-top surface microhardness. BTR was calculated as the ratio of the bottom- to the top-surface microhardness:

BTR = VMHb/VMHt(1)

In light-cured specimens irradiated from the top only, the light intensity and the degree of polymerization decrease with depth, so the bottom surface is the least cured region of the specimen; a BTR ≥ 0.80 therefore indicates that the intermediate layers are cured at least as well as the bottom surface. In self-cured Cention Forte (G1), polymerization is initiated chemically throughout the material, so the 0.80 criterion, developed for light-cured composites, was interpreted with caution. As BTR is an indirect, hardness-based index, it does not directly measure the degree of conversion.

#### 2.1.3. Diametral Tensile Strength Testing

Diametral tensile strength (DTS) was determined using disk-shaped specimens subjected to a compressive force applied parallel to their diameter until fracture. Specimens (4 mm high, 8 mm in diameter) were prepared according to ANSI/ADA Specification No. 27 [33] using a cylindrical silicone mold placed on a glass slide covered with a 50-μm polyester strip. After filling, the material was covered with a second polyester strip and glass slide and condensed under pressure. Tetric EvoCeram (G0) was placed in two 2-mm increments, each light-cured separately, while Cention Forte (G1 and G2) was placed in a single 4-mm increment.

The specimens were stored in light-proof containers with distilled and deionized water at 37 ± 1°C for 24 h to allow post-polymerization and material setting. The top and bottom surfaces were polished with silicon carbide instruments at 8000 rpm and rinsed with distilled water. Specimen dimensions were measured in triplicate using a digital micrometer (accuracy: 0.01 mm). Seven specimens were prepared per group (*n* = 7).

DTS was measured using a universal testing machine (Kason ETM504W-D, Jinan Kason Testing Equipment Co., Ltd.,Jinan, China) equipped with a force sensor (Chcontech CH-1B-50 KM, USA), at a crosshead speed of 0.5 mm/min. A compressive load was applied along the specimen diameter until fracture, and the maximum force (F) was recorded.

**Figure 2:**
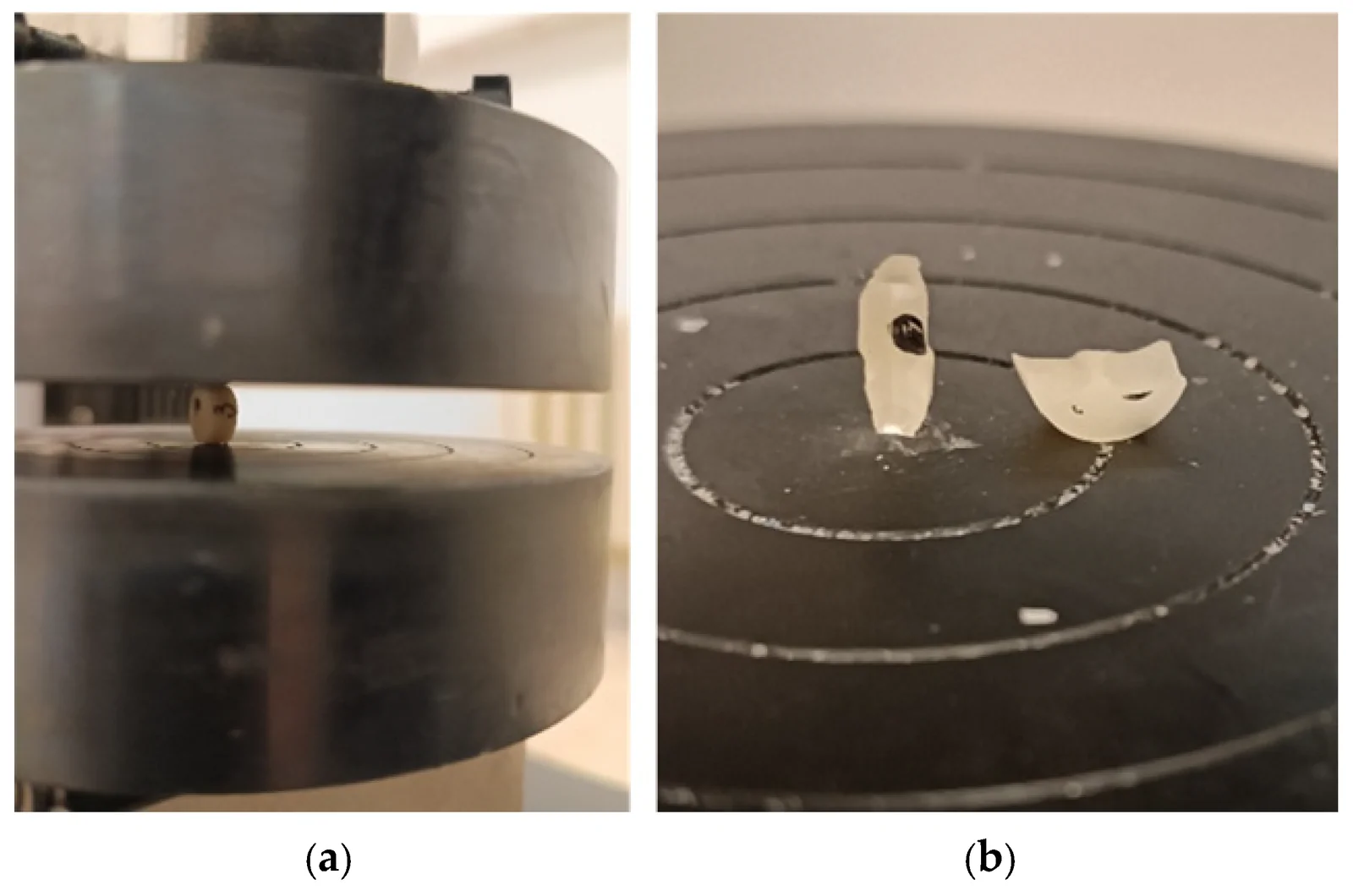
Diametral tensile strength test: (**a**) disk-shaped specimen positioned between the plates of the universal testing machine; (**b**) representative specimen after fracture, separated into fragments along the loaded diameter.

The failure criterion was specimen fracture, i.e., separation of the specimen into fragments along the loaded diameter, after which the load dropped. All specimens fractured along the loaded diameter. Plastic deformation at the loading contacts was not quantified. A specimen positioned in the testing machine and a representative fractured specimen are shown in Figure 2.

DTS was calculated using the following equation:

σ (DTS) = 2F/πdh

where σ(DTS) is the diametral tensile strength, F is the force at specimen fracture, d is the specimen diameter, h is the specimen height, and π is the mathematical constant (3.1416, used with full numerical precision). DTS is expressed in megapascals (MPa). Testing was performed at 21–23°C and 40–60% relative humidity, with seven specimens per group (*n* = 7).

#### 2.1.4. Compressive Strength Testing

Compressive strength (CS) was determined using cylindrical specimens (6 mm high, 4 mm in diameter) subjected to an axial load until fracture. Specimens were prepared in a two-part metal mold, covered with a polyester strip and glass slide, and condensed under pressure. Tetric EvoCeram (G0) was placed in three 2-mm increments, while Cention Forte (G1 and G2) was placed in a single 6-mm increment. Light curing followed the specified protocols.

Specimens were prepared at 21–23°C and 40–60% relative humidity, then stored in distilled and deionized water at 37 ± 1°C for 24 h in light-proof containers. The surfaces were polished at 8000 rpm with silicon carbide instruments, rinsed, and measured in triplicate using a digital micrometer (accuracy: 0.01 mm). Seven specimens were prepared per group (*n* = 7).

Compressive strength was measured using a universal testing machine (Kason ETM504W-D, Jinan Kason Testing Equipment Co., Ltd., China) equipped with a force sensor (Chcontech CH-1B-50 KM, USA). The crosshead speed was set to 0.5 mm/min, in accordance with ISO 9917-1 [34]. Cylindrical specimens were positioned vertically and compressed axially until fracture. The maximum force (F) was recorded electronically, and compressive strength was calculated from the measured specimen dimensions and fracture force using the following equation:

(2) $$\sigma \left(CS\right)=\frac{F}{\pi \cdot r^{2}}$$

where *σ*(CS) is the compressive strength, *F* is the force at specimen fracture, *r* is the specimen radius, and π is the mathematical constant (3.1416, used with full numerical precision). Compressive strength was expressed in megapascals (MPa), with seven specimens tested per group (*n* = 7).

### 2.2. Statistical Analysis

Statistical analyses were performed using IBM SPSS Statistics 30. Data normality was assessed using the Shapiro–Wilk test. Differences among the three materials were analyzed using one-way ANOVA. Variance homogeneity was assessed using Levene’s test. For variables with unequal variances (VMHt, VMHb, and BTR), Welch’s ANOVA with the Games–Howell post hoc test was applied, while standard ANOVA with Tukey’s post hoc test was used for DTS and CS. Statistical significance was set at *p*< 0.05.

Because filler content varied only between the two materials (two levels), it was not analyzed as a continuous variable. Instead, two planned comparisons were defined, in each of which only one factor differed: curing mode at identical material composition and filler content (G2 vs. G1) and material at an identical light-curing mode (G2 vs. G0). Differences were expressed as percentages of the reference group with 95% confidence intervals (delta method for the ratio of means, Welch–Satterthwaite degrees of freedom). In addition, the between-group sum of squares was partitioned by two orthogonal contrasts, material (G0 vs. the mean of G1 and G2) and curing mode (G1 vs. G2), to estimate the proportion of the between-group variance associated with each factor.

Mean differences between the groups were reported with 95% confidence intervals (Welch) and effect sizes (Hedges g). The sample size of seven specimens per group was chosen in accordance with comparable in vitro studies [23,24,25,26,27,28,29,30,31] and exceeds the minimum of five specimens per group required by ISO 9917-1. No a priori power analysis was performed; a sensitivity analysis showed that, with *n* = 7 per group, a two-sided significance level of 0.05 and 80% power, standardized differences (Cohen d) of approximately 1.65 or larger could be detected.

## 3. Results

Descriptive statistics and the normality of the distributions of the investigated mechanical properties are presented in Table 2.

Welch’s ANOVA revealed statistically significant differences among the materials in Vickers microhardness measured on the top surface (VMHt), F(2, 8.93) = 1558.13, *p*< 0.001, and the bottom surface (VMHb), F(2, 8.50) = 2253.34, *p*< 0.001. The Games–Howell post hoc test confirmed statistically significant differences between all material pairs for both parameters (*p*< 0.001). The highest VMHt and VMHb values were recorded for self-curing Cention Forte, followed by Tetric EvoCeram, whereas light-cured Cention Forte exhibited the lowest values. A statistically significant difference was also found for the bottom-to-top ratio (BTR), F(2, 8.22) = 30.40, *p*< 0.001. The Games–Howell test revealed a significant difference between Tetric EvoCeram and self-curing Cention Forte (*p*< 0.001), while the remaining pairwise differences were not statistically significant (*p* = 0.118 and *p* = 1.000).

One-way ANOVA revealed statistically significant differences among the three investigated materials in diametral tensile strength (DTS), F(2, 18) = 141.66, *p*< 0.001, and compressive strength (CS), F(2, 18) = 80.78, *p*< 0.001. Tukey’s post hoc test showed that all materials differed significantly from one another. The highest DTS value was recorded for light-cured Cention Forte (M = 39.7 MPa), whereas the lowest was observed for Tetric EvoCeram (M = 25.5 MPa). In contrast, Tetric EvoCeram exhibited the highest CS value (M = 311.2 MPa), while self-curing Cention Forte had the lowest value (M = 236.0 MPa).

Mean differences between the groups with 95% confidence intervals and effect sizes are presented in Table 3.

**Figure 3:**
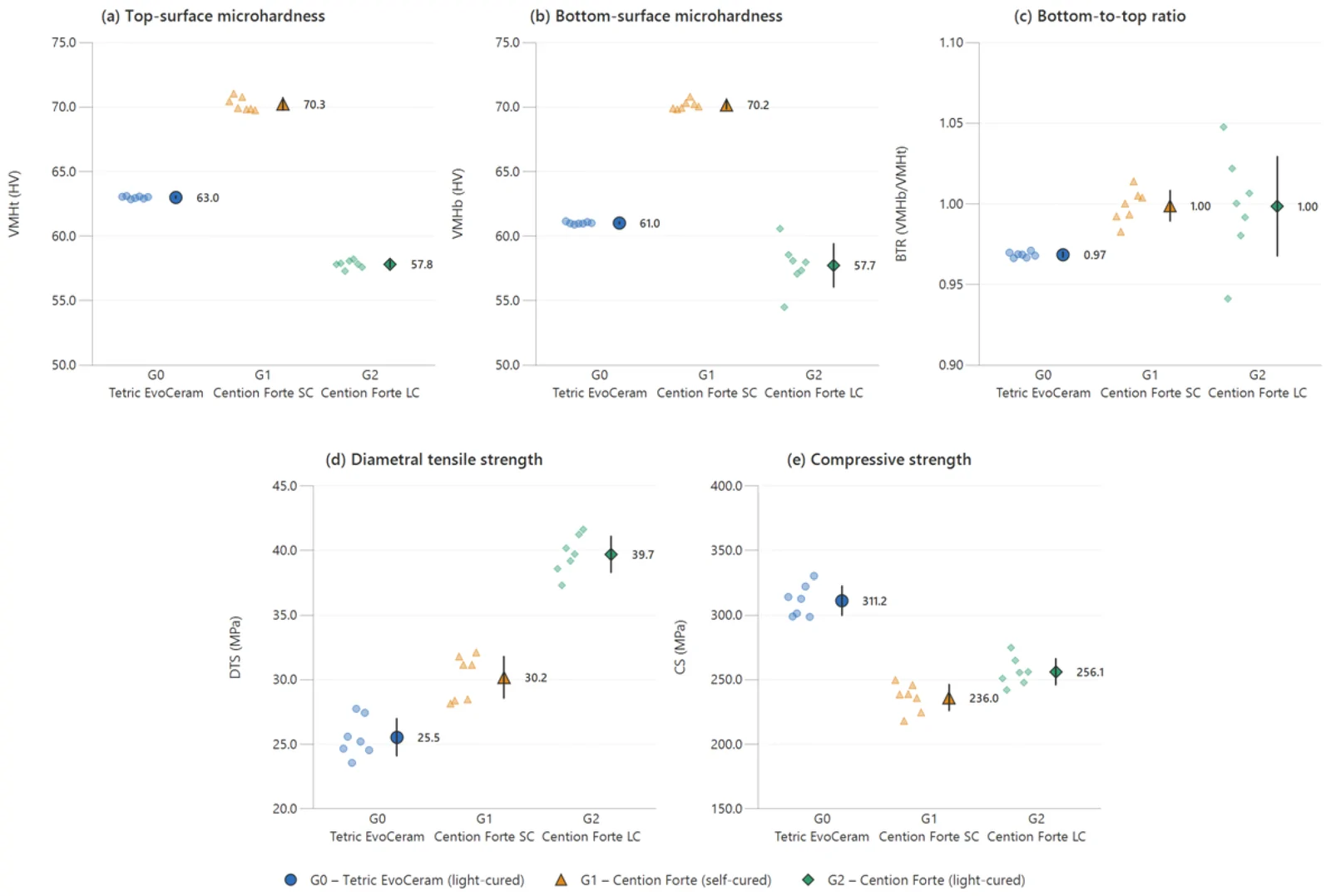
Mechanical properties of the tested materials: (**a**) top-surface Vickers microhardness (VMHt), (**b**) bottom-surface Vickers microhardness (VMHb), (**c**) bottom-to-top microhardness ratio (BTR), (**d**) diametral tensile strength (DTS) and (**e**) compressive strength (CS). Small symbols represent individual specimens (*n* = 7 per group); large symbols with error bars represent means with 95% confidence intervals.

Individual values and means with 95% confidence intervals of all investigated properties are presented in Figure 3.

**Figure 4:**
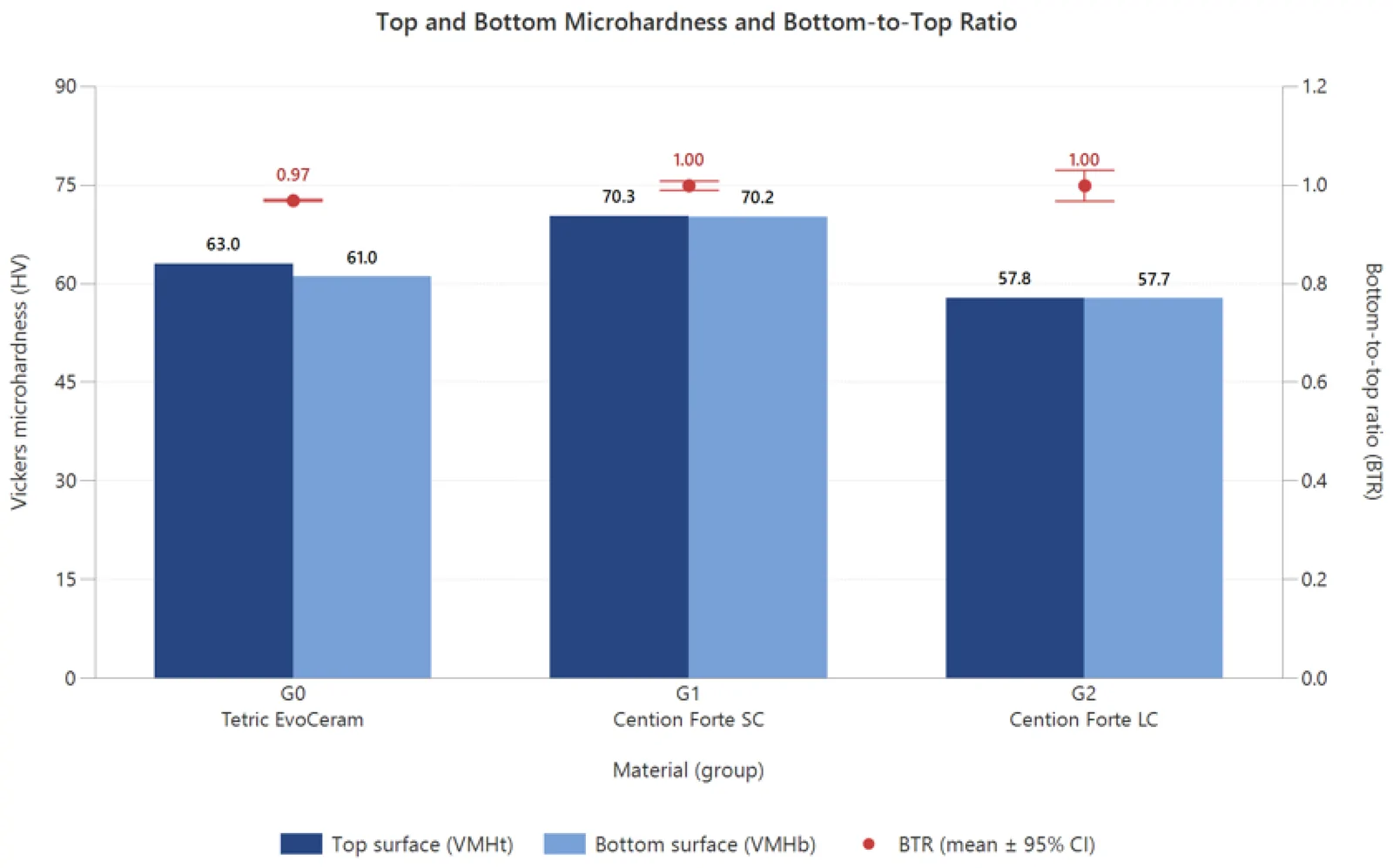
Top- and bottom-surface Vickers microhardness (bars) and bottom-to-top microhardness ratio (BTR, right axis; mean ± 95% CI) of the tested materials.

The mean BTR values were 0.97 for Tetric EvoCeram and 1.00 for both self-cured and light-cured Cention Forte (Table 2). All groups showed BTR values above 0.80, the generally accepted criterion of adequate curing [35]. For self-cured Cention Forte, in which polymerization is initiated chemically throughout the material, this criterion was interpreted with caution; the BTR of approximately 1.00 reflects comparable microhardness of the top and bottom surfaces (Figure 4).

**Figure 5:**
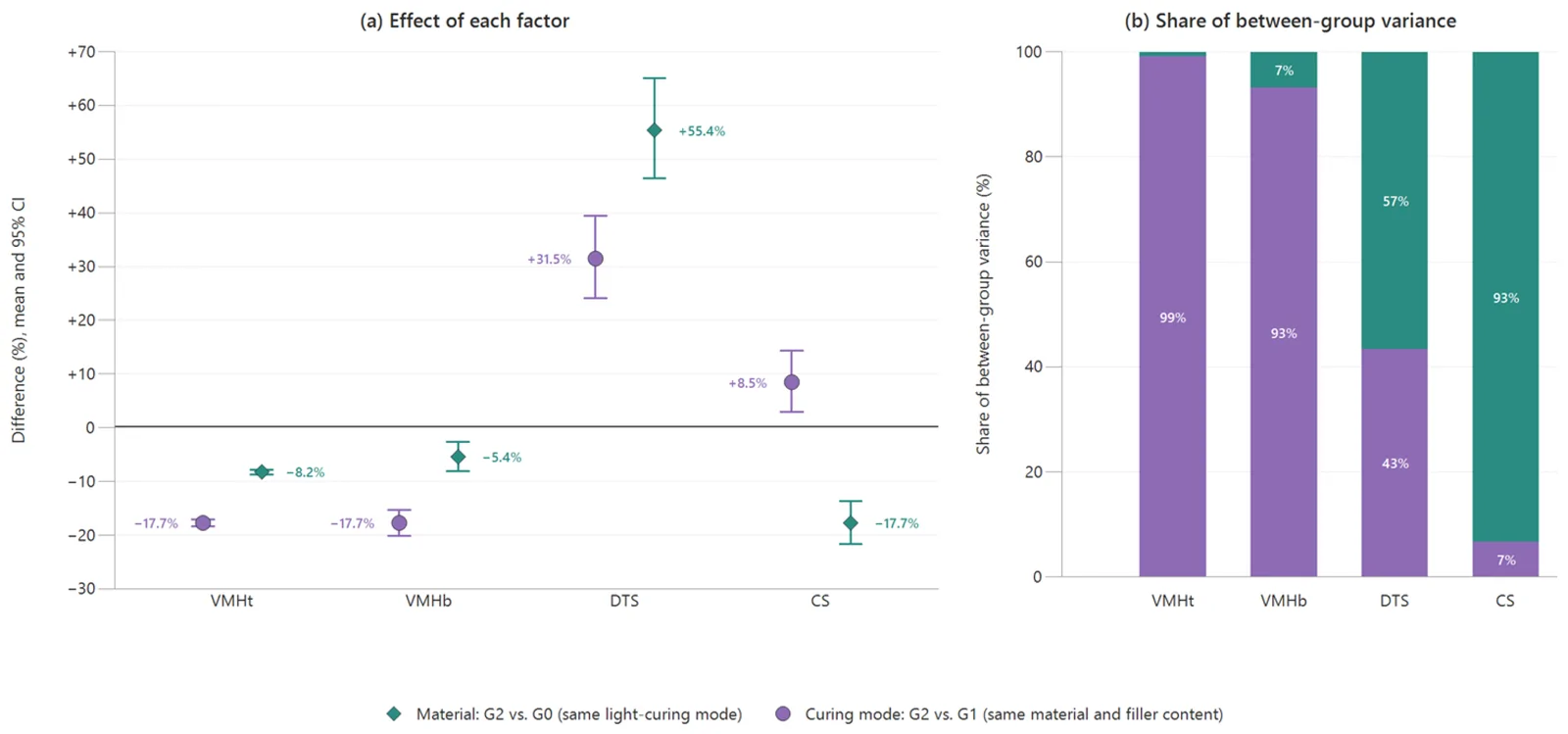
Separation of the effects of curing mode and material. (**a**) Percentage differences (mean and 95% CI) for curing mode (light-cured vs. self-cured Cention Forte; same composition and filler content) and for material (Cention Forte vs. Tetric EvoCeram, both light-cured). (**b**) Proportion of the between-group variance associated with curing mode (G1 vs. G2) and material (G0 vs. mean of G1 and G2).

Because filler content varied only between the two materials and G1 and G2 have identical composition, the effects of curing mode and material were separated by planned comparisons (Table 4, Figure 5). At identical composition and filler content, light curing of Cention Forte reduced VMHt and VMHb by 17.7% but increased DTS by 31.5% and CS by 8.5%. At an identical light-curing mode, Cention Forte showed lower VMHt (−8.2%), VMHb (−5.4%) and CS (−17.7%) but a higher DTS (+55.4%) than Tetric EvoCeram. Curing mode accounted for 99.1% and 93.1% of the between-group variance in VMHt and VMHb, material for 93.4% of the variance in CS, while both factors contributed to DTS (curing mode 43.4%, material 56.6%).

## 4. Discussion

This study evaluated the mechanical properties of the alkasite material Cention Forte (G1 and G2), using two polymerization protocols, and the conventional nanohybrid composite Tetric EvoCeram.Cention Forte is a direct restorative material based on urethane dimethacrylate (UDMA), with dual-curing capability (light/self-curing). It is classified as bioactive due to the release of fluoride, calcium, and phosphate ions. Hydroxyl ion release also alkalizes the surrounding environment, potentially contributing to caries prevention. Studies have reported comparable or superior physical and mechanical properties of alkasites relative to commonly used restorative materials [36].

Cention Forte was evaluated in two groups: G1, self-cured, and G2, light-cured according to the manufacturer’s instructions using 2000 mW/cm^2^ for 10 s.Comparison of top and bottom microhardness revealed no significant differences for self-cured (*p* = 0.771) or light-cured (*p* = 0.91) Cention Forte. This was expected given its dual-curing mechanism, whereby self-curing continues after light exposure.Both groups showed nearly identical top- and bottom-surface microhardness (BTR ≈ 1.00), well above the generally accepted threshold of 0.80 [32], indicating effective curing of the bottom surface of the 4-mm specimens. In light-cured specimens, the bottom surface is the least cured region, so the intermediate layers are cured at least as well; in self-cured specimens, polymerization is initiated chemically throughout the material. As BTR is an indirect, hardness-based index, direct measurement of the degree of conversion would provide additional confirmation.

Compared with Tetric EvoCeram, self-cured Cention Forte (G1) showed significantly higher top- and bottom-surface microhardness and diametral tensile strength, but significantly lower compressive strength. Because the materials performed differently in different tests and no criterion for an overall ranking was defined, none of the materials can be considered superior overall. These findings are consistent with previous studies: Sardana et al. reported higher surface microhardness of alkasite than conventional nanohybrid composite [37], Paul et al. found higher DTS for Cention than for nanohybrid composite and conventional glass-ionomer cement [38], and Mazumdar et al. reported the highest Vickers microhardness for Cention among the tested restorative materials [39].

Turkistani et al. found significantly lower microhardness of Cention Forte than conventional nanohybrid composite and resin-modified glass-ionomer cement (Fuji II LC), with a marked decrease following acid exposure [40]. These findings are consistent with our results for light-cured Cention Forte. Naz et al. reported greater resistance to simulated chewing forces for Cention N than for conventional glass-ionomer cement (Fuji IX) and composite (Filtek Z250) [41]. Conversely, Panpisut et al. found the highest flexural strength in conventional composite, although Cention N outperformed resin-modified glass-ionomer cements Fuji II LC and Riva LC [42]. Cention’s high diametral tensile and flexural strength may be attributed to its UDMA content, which improves polymer network density and depth of polymerization [29].

Iftikhar et al. found that conventional composites had the highest compressive and diametral tensile strength, while conventional glass-ionomer cement (GIC) had the lowest. Cention showed mechanical properties comparable to conventional composites [43]. These findings are consistent with our results for compressive strength. Similarly, Sujith et al. reported the highest compressive strength for conventional composite and the lowest for GIC, while alkasite showed the highest flexural strength [44]. Their findings regarding compressive and diametral tensile strength are consistent with our results.

Lalović et al. found that Cention Forte had significantly higher flexural and diametral tensile strength than hybrid and conventional GICs. Bulk-fill composite showed the highest compressive strength, although the difference from Cention Forte was not significant [45]. In our study, Tetric EvoCeram had significantly higher compressive strength(range 298.7–330.3 MPa; mean 311.2 MPa) than Cention Forte (overall range 218.2–274.9 MPa; G1 mean 236.0 MPa, G2 mean 256.1 MPa). Similarly, Justen et al. reported that Cention has physical and mechanical properties comparable to composites and superior to GICs, supporting its potential use in permanent restorations [46].

Light-cured Cention Forte (G2) showed lower microhardness but higher compressive and diametral tensile strength than the self-cured material. It exhibited the highest DTS of the three materials, whereas the lowest CS was recorded for self-cured Cention Forte (G1). Cention’s ability to release and recharge calcium and phosphate ions may enhance its surface microhardness [36]. However, Turkistani et al. reported lower microhardness than conventional nanohybrid composite, with a significant decrease following acid exposure [40]. Cention’s high diametral tensile and flexural strength may be attributed to its UDMA content, which improves polymer network density and depth of polymerization [29].

The spherical filler particles in Cention N allow higher filler loading and may improve compressive strength by reducing stress concentration at sharp edges [47]. Unlike GICs and resin-modified GICs, Cention lacks polyacrylic acid derivatives, resulting in weaker chemical bonding to tooth structure; therefore, an adhesive primer is required before placement [36]. Overall, Cention exhibits favorable physical and mechanical properties comparable to composites. However, further long-term clinical studies are needed to assess its mechanical performance and the effects of environmental factors, such as saliva, toothpaste, and beverages.

Comparison of self-cured and light-cured Cention Forte. Self-cured Cention Forte (G1) showed higher top microhardness (VMHt) than light-cured Cention Forte (G2). This may be partly related to specimen preparation, because only the self-cured specimens were kept under a static load of 500 g for 6 min during setting, which may have increased their compaction; as the light-cured groups were not subjected to the same load, the contribution of this factor cannot be quantified, and the difference between G1 and G2 should be interpreted with caution. Differences may also reflect the effect of light curing on mechanical properties. Similarly, Kaptan et al. reported significantly lower flexural strength in light-cured than in self-cured Cention, suggesting that rapid light-induced setting may inhibit further self-curing [21].

In our study, light curing improved the compressive and diametral tensile strength of Cention Forte. Similarly, de Araújo-Neto et al. reported increased flexural strength in light-cured compared with self-cured Cention N [35].In contrast to Fousiya et al., who found no effect of light curing on the compressive or flexural strength of Cention N [22], light curing significantly increased the compressive strength of Cention Forte in the present study (G2 vs. G1, +8.5%), which may reflect the different products (Cention N vs. Cention Forte) and test conditions.

Burrer et al. found that self-cured Cention had a significantly lower degree of conversion and the slowest polymerization rate among the tested dual-cure materials [48]. Since a high degree of conversion is essential for material performance and mechanical properties [49], some authors advise against using Cention in self-curing mode due to its inferior chemical and mechanical properties [48,50]. Marović et al. reported a higher degree of conversion in light-cured than in self-cured Cention, suggesting that light exposure improves monomer-to-polymer conversion [50]. Similarly, Negovetic Mandić et al. found significantly lower microhardness and flexural strength in self-cured Cention Forte than in light-cured specimens [51]. Regarding microhardness, these findings differ from our results, possibly due to differences in specimen preparation for microhardness testing.

Cention Forte and Tetric EvoCeram differ not only in filler content (78.4 vs. 75–76 wt%) but also in resin matrix, filler chemistry and particle characteristics, curing mechanism and placement technique. Therefore, the difference between the two light-cured groups (G0 and G2) reflects the combined effect of material composition at the same curing mode and cannot be attributed to filler content alone, whereas the comparison of G1 and G2, which have an identical composition, isolates the effect of curing mode. In the present study, curing mode accounted for most of the between-group variance in microhardness (VMHt 99.1%, VMHb 93.1%), material for most of the variance in compressive strength (93.4%), and both factors contributed to DTS (Table 4, Figure 5).

A higher filler volume fraction is generally associated with higher hardness of resin composites [52]. Although Cention Forte contains approximately 4.3 vol% more filler than Tetric EvoCeram, at the same light-curing mode it showed lower microhardness (−8.2%) and lower compressive strength (−17.7%). The direction of these differences is opposite to that expected from filler quantity alone, indicating that other material characteristics, such as filler type and reactivity, particle size and shape, resin matrix composition, filler–matrix coupling and degree of conversion, have a greater influence on these properties than filler content. Because filler content varied only between two commercial materials of different composition, its independent effect could not be determined in the present study.

This study has several limitations. First, only two commercial materials, and therefore only two filler-content levels, were compared, so the independent effect of filler content could not be determined. Second, BTR is an indirect, hardness-based index, and the degree of conversion was not measured directly. Third, only the self-cured specimens were kept under a static load of 500 g during setting, whereas the light-cured specimens were condensed manually. Fourth, the 6-mm Cention Forte compressive strength specimens were light-cured from the top surface only, so the curing of their deeper parts relied mainly on the chemical setting reaction. Fifth, plastic deformation at the loading contacts during DTS testing was not quantified. Finally, with seven specimens per group, the study could detect only large differences (d ≥ 1.65); the smallest significant difference (CS, G1 vs. G2) was close to this threshold and should be interpreted with caution.

Taken together, these findings suggest that Cention has mechanical properties comparable to those of composites, while its single-increment application and remineralizing potential may offer advantages in restorative dentistry. Further laboratory and long-term clinical studies are needed to confirm these findings.

## 5. Conclusions

Within the limitations of this in vitro study, the following conclusions can be drawn:

1. Self-cured Cention Forte exhibited the highest top- and bottom-surface Vickers microhardness, significantly exceeding Tetric EvoCeram, whereas light-cured Cention Forte showed the lowest values.

2. Both self-cured and light-cured Cention Forte exhibited the expected bottom-to-top hardness ratio (BTR) of approximately 1.00, while Tetric EvoCeram showed a BTR of 0.97. These values indicate comparable hardness between the bottom and top surfaces, suggesting effective curing at the bottom surface. However, as BTR is an indirect, hardness-based index, direct measurement of the degree of conversion would provide additional confirmation of polymerization efficiency.

3. Light curing increased the diametral tensile strength and compressive strength of Cention Forte compared with self-curing, but reduced its surface microhardness. Light-cured Cention Forte showed the highest diametral tensile strength of all tested materials.

4. Tetric EvoCeram exhibited the highest compressive strength. Cention Forte values were 17.7–24.2% lower.

5. Curing mode accounted for most of the between-group variance in microhardness, whereas material composition dominated compressive strength, and both factors contributed to diametral tensile strength. Because only two filler-content levels were available, the independent effect of filler content could not be determined.

6.Compared with the conventional resin composite, Cention Forte showed higher (self-cured) or lower (light-cured) microhardness, higher diametral tensile strength and lower compressive strength; its performance therefore depended on the property and the curing mode. Its single-increment application and ion-releasing potential may offer clinical advantages. Long-term clinical studies are needed to confirm these findings.

## Figures and Tables

**Table 1:** Materials used in the study.

Material Type	Brand Name	Setting Type	Manufacturer	Shade	Polymerization Mode	Filler Content (wt%/vol%)
Alkasite-based resin composite	Cention^®^ Forte (CF)	Self-curing (CFSC) G1	Ivoclar Vivadent AG Schaan, Liechtenstein	A2	Self-curing	78.4/57.6–59
		Light-curing (CFLC) G2			Light curing 2000 mW/cm^2^, 2 × 5 s (Bluephase PowerCure Ivoclar Vivadent AG Schaan, Liechtenstein)	
Nanohybrid composite	Tetric^®^ EvoCeram (TEC)	Light-curing G0	Ivoclar Vivadent AG Schaan, Liechtenstein	A2	Light curing 1200 mW/cm^2^, 10 s (Bluephase PowerCure Ivoclar Vivadent AG Schaan, Liechtenstein)	75–76/53–55

**Table 2:** Mechanical Properties: descriptive statistics and normality of distribution.

Variable Group Material			N	Mean	SD	SE	95% CI		Min	Max	Skewness			Kurtosis			Shapiro–Wilk		
							Lower	Upper			Stat.	SE	Zsk	Stat.	SE	Zku	W	df	*p*
G0 Tetric EvoCeram			7	63.0	0.1	0.0	62.9	63.1	62.9	63.1	−0.48	0.79	−0.61	−1.15	1.59	−0.72	0.945	7	0.686
VMHt (HV)	G1	Cention Forte Self Cure	7	70.3	0.5	0.2	69.8	70.7	69.8	71.1	0.74	0.79	0.94	−1.41	1.59	−0.89	0.843	7	0.106
	G2	Cention Forte Light Cure	7	57.8	0.3	0.1	57.5	58.1	57.3	58.2	−0.62	0.79	−0.78	0.45	1.59	0.28	0.963	7	0.847
VMHb (HV)	G0	Tetric EvoCeram	7	61.0	0.1	0.0	60.9	61.1	60.9	61.2	0.52	0.79	0.66	0.13	1.59	0.08	0.946	7	0.693
	G1	Cention Forte Self Cure	7	70.2	0.3	0.1	69.9	70.5	69.8	70.8	1.34	0.79	1.70	1.73	1.59	1.09	0.884	7	0.244
	G2	Cention Forte Light Cure	7	57.7	1.8	0.7	56.0	59.4	54.5	60.6	−0.41	0.79	−0.52	1.98	1.59	1.25	0.942	7	0.659
BTR	G0	Tetric EvoCeram	7	0.97	0.002	0.001	0.967	0.970	0.966	0.971	0.26	0.79	0.33	−0.62	1.59	−0.39	0.962	7	0.838
	G1	Cention Forte Self Cure	7	1.00	0.010	0.004	0.989	1.008	0.983	1.014	−0.16	0.79	−0.20	−0.11	1.59	−0.07	0.983	7	0.971
	G2	Cention Forte Light Cure	7	1.00	0.033	0.013	0.968	1.029	0.941	1.048	−0.39	0.79	−0.49	0.99	1.59	0.62	0.981	7	0.965
DTS (MPa)	G0	Tetric EvoCeram	7	25.5	1.5	0.6	24.1	27.0	23.6	27.8	0.53	0.79	0.67	−0.96	1.59	−0.60	0.917	7	0.448
	G1	Cention Forte Self Cure	7	30.2	1.8	0.7	28.6	31.8	28.2	32.1	−0.24	0.79	−0.30	−2.53	1.59	−1.59	0.817	7	0.059
	G2	Cention Forte Light Cure	7	39.7	1.5	0.6	38.3	41.1	37.3	41.6	−0.26	0.79	−0.33	−0.50	1.59	−0.31	0.977	7	0.941
CS (MPa)	G0	Tetric EvoCeram	7	311.2	12.2	4.6	300.0	322.5	298.7	330.3	0.45	0.79	0.57	−1.18	1.59	−0.74	0.906	7	0.367
	G1	Cention Forte Self Cure	7	236.0	11.2	4.2	225.7	246.3	218.2	249.9	−0.57	0.79	−0.72	−0.51	1.59	−0.32	0.943	7	0.663
	G2	Cention Forte Light Cure	7	256.1	11.0	4.1	245.9	266.2	242.1	274.9	0.69	0.79	0.87	0.22	1.59	0.14	0.961	7	0.826

Variables: VMHt—Vickers microhardness of the top surface; VMHb—Vickers microhardness of the bottom surface; BTR—bottom-to-top microhardness ratio (VMHb/VMHt); DTS—diametral tensile strength; CS—compressive strength. Values: N—number of specimens; Mean—arithmetic mean; SD—standard deviation; SE—standard error; 95% CI—95% confidence interval of the mean (Lower/Upper—lower and upper bound); Min/Max—minimum and maximum value; Stat.—skewness/kurtosis statistic; Zsk, Zku—z-values of skewness and kurtosis (statistic/SE); W—Shapiro–Wilk statistic; df—degrees of freedom; *p*—significance.

**Table 3:** Mean differences between groups with 95% confidence intervals (Welch, unadjusted) and effect sizes (Hedges g).

Property	Comparison	Mean Difference	95% CI	Hedges g
VMHt (HV)	G1 − G0	+7.2	6.8 to 7.7	18.1
	G2 − G0	−5.2	−5.5 to −4.9	−21.6
	G2 − G1	−12.4	−12.9 to −11.9	−27.3
VMHb (HV)	G1 − G0	+9.1	8.8 to 9.5	34.8
	G2 − G0	−3.3	−5.0 to −1.6	−2.4
	G2 − G1	−12.4	−14.1 to −10.7	−8.9
BTR	G1 − G0	+0.030	0.021 to 0.040	3.9
	G2 − G0	+0.030	−0.001 to 0.061	1.2
	G2 − G1	0.000	−0.031 to 0.031	0.0
DTS (MPa)	G1 − G0	+4.6	2.7 to 6.6	2.6
	G2 − G0	+14.2	12.4 to 15.9	8.7
	G2 − G1	+9.5	7.6 to 11.4	5.5
CS (MPa)	G1 − G0	−75.2	−88.9 to −61.6	−6.0
	G2 − G0	−55.2	−68.7 to −41.6	−4.5
	G2 − G1	+20.1	7.2 to 33.0	1.7

G0—Tetric EvoCeram; G1—self-cured Cention Forte; G2—light-cured Cention Forte. Only the BTR differences G2 − G0 and G2 − G1 include zero, consistent with the post hoc tests.

**Table 4:** Effects of curing mode and material on the mechanical properties.

Property	Curing Mode: G2 vs. G1, % (95% CI)	*p*	Material: G2 vs. G0, % (95% CI)	*p*	Share of Between-Group Variance: Curing/Material (%)
VMHt	−17.7 (−18.3 to −17.1)	<0.001	−8.2 (−8.7 to −7.8)	<0.001	99.1/0.9
VMHb	−17.7 (−20.1 to −15.3)	<0.001	−5.4 (−8.1 to −2.6)	0.003	93.1/6.9
DTS	+31.5 (24.1 to 39.4)	<0.001	+55.4 (46.4 to 65.1)	<0.001	43.4/56.6
CS	+8.5 (2.9 to 14.4)	0.005	−17.7 (−21.6 to −13.7)	<0.001	6.6/93.4

Curing mode: light-cured vs. self-cured Cention Forte (identical composition and filler content). Material: Cention Forte vs. Tetric EvoCeram, both light-cured. *p* values from Welch tests; all remain <0.05 after Bonferroni correction for the two comparisons. The share of between-group variance was obtained from two orthogonal contrasts (material: G0 vs. mean of G1 and G2; curing mode: G1 vs. G2).

## Data Availability

The raw data supporting the conclusions of this article will be madeavailableby the authors on request.
